# Supplementary material for: Uncovering Offshore Financial Centers: Conduits and Sinks in the Global Corporate Ownership Network
Source: Sci Rep. 2017 Jul 24;7:6246. doi: 10.1038/s41598-017-06322-9 (PMC5524793; doi:10.1038/s41598-017-06322-9)
Supplement: Supplementary file 1 — Supplementary Information [file 41598_2017_6322_MOESM1_ESM.pdf]

Uncovering Offshore Financial Centers: Conduits and Sinks in the Global Corporate Ownership Network

Javier Garcia-Bernardo[1], Jan Fichtner[1], Frank W. Takes[1,2], and Eelke M. Heemskerk[1]

[1]CORPNET, Amsterdam Institute for Social Science Research, University of Amsterdam, Nieuwe Achtergracht 166, 1018 WV, Amsterdam, The Netherlands

[2]LIACS, Department of Computer Science, Leiden University, Niels Bohrweg 1, 2333 CA, Leiden, The Netherlands

*{garcia,j.r.fichtner,takes,e.m.heemskerk}@uva.nl

Supplementary

Supplementary Methods

Cities in Isle of Man (IM), Jersey (JE) and Guernsey (GG)

Companies under the country code of the United Kingdom in the following cities were assigned to IM, JE and GG:

Table S1. Cities under the GB country code assigned to IM, JE and GG

Code	Cities
IM	DOUGLAS, RAMSEY, CASTLETOWN, ONCHAN, PEEL, BRADDAN, PORT ERIN, BALLASALLA, PORT SAINT MARY, LAXEY, SAINT JOHN'S, KIRK MICHAEL, SANTON
JE	SAINT HELIER, JERSEY, SAINT CLEMENT, SAINT SAVIOUR, SAINT PETER, SAINT MARTIN, SAINT LAWRENCE, SAINT OUEN, TRINITY, SAINT JOHN,SAINT MARY, ST HELIER, GROUVILLE, ST. HELIER, ST. HELIER, JERSEY
GG	GUERNSEY, ST PETER PORT, ST. PETER PORT, ST. PETER PORT, GUERNSEY, SAINT PETER PORT

Deconsolidation of financial statements

Deconsolidation takes place in two steps. In the first step, all companies under the same global ultimate owner are grouped and the ownership structure constructed. Starting from the bottom of the tree (the small subsidiaries) we tracked up the subsidiaries of companies with consolidated accounts. Moreover, we considered a company A subsidiary of a company B (with consolidated accounts) if they shared the same global ultimate owner and their values of revenue and number of employees were within 25% of each other, even when no ownership link was recorded in the database. We then iteratively (from the bottom of the tree to the root) subtracted the number of employees and the operating revenue of the subsidiaries.

In the second step, all companies with more than 1000 employees were grouped together. We considered company A subsidiary of a company B (with consolidated accounts) if their values of revenue were within 25% of each other. We then iteratively substracted the operating revenue of the subsidiaries. This approach corrects for duplicated information among large companies[1].

Normalization of ownership

Since the information is collected by different country-level agencies and merged by Orbis, the sum of the stakes of the shareholders do not always add up to 100%. We corrected by collecting all direct ownership stakes. When the sum of the direct ownership stakes was below 100% we added total ownership up to 100%, when it was above we normalized the ownership to sum up to 100%.

Mathematical formulation of country chains

The paper provides an explanation of the process from ownership links to country chains based on the different construction steps. Here we outline the theoretical definitions of the concepts obtained in each of these steps.

In the global corporate ownership network $N = (F, E)$, firms are represented as a set F of size $n = |F|$. The set of ownership relations $E \subseteq F \times F$ contains a total of $m = |E|$ pairs (i, j) indicating that there is a directed *ownership* relation between firms $i, j \in F$. Here, firm j owns i and thus value may flow from i to j. The link weight $w(i, j) \in [0; 1]$ or in short w_{ij} represents the ownership percentage of a relation $(i, j) \in E$. For example, the value of w_{ij} is equal to 0 for non-existing links, equal to 1 for fully owned subsidiaries of j and 0.3 in case of 30% ownership. The value of a node i, denoted $R(i)$ or in short R_i, represents the (always positive) value of firm i. Here we use the revenue of the firm.

Multiple ownership relations may together form an *ownership path*: an ordered sequence of firms in which each subsequent pair of firms is connected through an ownership link. So, for a path p of length $\ell = |p|$ with firms $p = (v_1, v_2, \ldots, v_\ell)$ it holds that $(v_i, v_{i+1}) \in E$ for $1 \leq i < \ell$. For brevity, in the paper such as a path is denoted $v_1|v_2|\ldots|v_\ell$. A *simple* path has no repeated nodes, i.e., no cycles. The notion of *multiplicative ownership* $w(p)$ or in short w_p models the ownership weight relation $w(v_i, v_\ell)$ along a particular ownership path $p = (v_1, v_2, \ldots, v_\ell)$ of length $\ell = |p|$ as the multiplication of weights of the links between the subsequent nodes in the path, i.e.,

$$w_p = w(p) = \prod_{i=1}^{\ell-1} w(v_i, v_{i+1})$$

The *value* $V(p)$ of a path p, in short V_p, is defined as the value that flows from the first to the last node in the path, i.e., the product of the value of this first node and the multiplicative ownership of the path:

$$V_p = V(p) = R_{v_1} \cdot w_p$$

An *ownership chain* of a firm v is an ownership path p which satisfies four criteria: it starts at node v, it is a simple path (has no repeated nodes), it has a multiplicative ownership value of at least threshold θ, i.e., $w_p \geq \theta$ and is maximal in length, i.e., cannot be extended by adding another node. Experiments with different values θ are discussed in the section 'Sensitivity analysis' of

the Supplementary Information. A node typically starts more than one ownership chain, and the set of all ownership chains starting at node $v \in F$ is denoted $C(v)$ or in short C_v. Ultimately, C represents the set of all ownership chains in the network:

$$C = \bigcup_{v \in F} C_v$$

Each chain $p \in C$ in the set of ownership chains is in fact a path of length $\ell = |p|$. From an ownership chain, we can generate all possible subpaths of length $2, 3, \ldots, \ell$, which together we call the set of *ownership chunks*, denoted H. The set of ownership chunks of length x is denoted H^x. Each chunk $q \in H$ has an associated value $V^p(q)$ or in short V_q^p. This value depends on the value of the first node in the ownership chain p that chunk q originated from, as well as the path followed from that node to chunk q.

For each node v, a function $\phi(v) \rightarrow I$ indicates the country $c \in I$ in which firm v is based. The function can be applied to both paths and individual nodes. For each previously obtained chunk $q = (v_1, v_2, \ldots, v_\ell)$, we create a *country chain* in two steps. First, we map each node in the chunk to its respective country, obtaining:

$$\phi(q) = (\phi(v_1), \phi(v_2), \ldots, \phi(v_\ell))$$

Note that in the main text of the paper, for brevity when we talk about country chains we use the ISO 2-letter country codes combined with the shorthand notation discussed above, e.g., $NL|LU|KY$. Second, we merge any two subsequent nodes of the same country v_i, v_{i+1} in a mapped chunk $\phi(q)$, i.e., if it holds that $\phi(v_i) = \phi(v_{i+1})$, replace this pair by $\phi(v_i)$. This results in country chain g. The valuation function $V^\phi(g)$ of a country chain $g \in G$ sums the weights of the ownership chunks that map to this particular country chain. For brevity, in the main text of the paper we again use V_g when it is clear from the context that we consider a country chain g. Note that as a result of the second step, the length of a resulting country chain may be shorter than the length of the originating ownership chunk. Furthermore, multiple ownership chunks may result in the same country chain. Applying this process to all ownership chunks in H results in the full set of *country chains* G. Analogously to before, we denote the set of country chains of length x as G^x. These chains are the basis for the definitions of sink-OFC and conduit-OFC centrality proposed in the main text of the paper.

Comparison of our data with Foreign Direct Investment (FDI)

FDI reflects controlling ownership stakes in all the companies in one country by all the companies located in another country. In order to further assess the quality of our data, we compared the value of transnational ownership ties of firms from a particular country against the foreign direct investment (FDI) of that country, as provided by the IMF. Since some countries systematically under-report inward FDI, we kept for each country the maximum value between the value reported by the country, and the sum of outward FDI to that country as reported by the counterpart economies. The weighted ownership matches well with FDI data (Figure S1).

Null model for Figure 3

Companies own stakes of other firms across the world. When these stakes are aggregated at the country level, we obtain a fully connected network where the weight of the link corresponds to the sum of value flowing between the pair of countries. In order

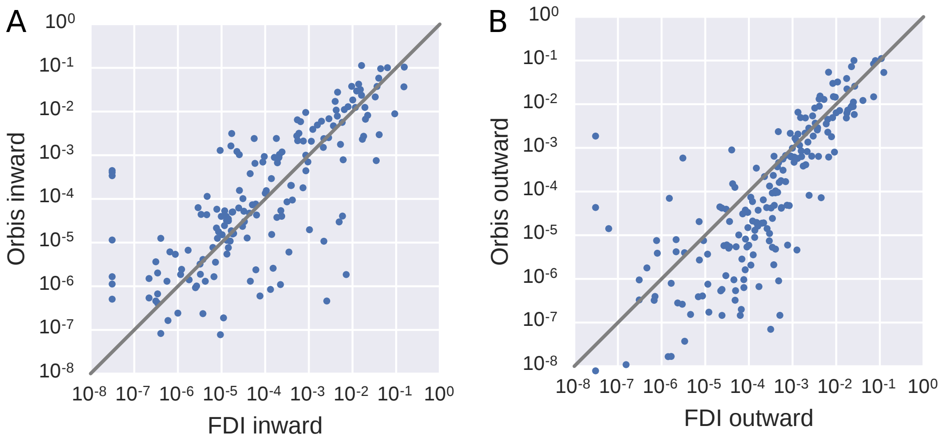

Figure S1. FDI vs aggregated ownership data. (A) Fraction of revenue ending in country c versus fraction of inward FDI associated to country c. (B) Fraction of revenue starting in country c vs. fraction of outward FDI associated to country c. Since FDI reflects only controlling shareholdings, only chains with more than 50% ownership were included.

to keep only significant links, we created a null model where the weight between two countries was set to the product of the GDP of both countries. We kept only those edges with a weight 10 times larger than in the null model – after normalizing both networks to have the same sum of edge weights.

Sector specialization

Starting from the global corporate ownership chains of size three (G^3) we mapped each company to its corresponding sector code (NACE Rev. 2) as provided by Orbis. We then grouped all sectors according to their dominant position in chains of size three: the first position (source), second (conduit) and third (sink), finding six categories: only source, only conduit, only sink, source+conduit, source+sink, conduit+sink and source+conduit+sink, by using the criteria in Table S2.

Finally, the weight of a sector within a category (e.g., sink) was calculated as the sum of the value of the chains where the sector participates in its category (sink) minus the sum of the value of the chains where the sector participates in other categories (conduit or source). The weight was further normalized by the sum of the value of companies that participate in the network in such category.

Table S2. Sector classification by category

CAT	Criteria
SO	$> \frac{1}{2}$ of all G^3 containing a given sector contain it in the source position.
CO	$> \frac{1}{2}$ of all G^3 containing a given sector contain it in the conduit position.
SI	$> \frac{1}{2}$ of all G^3 containing a given sector contain it in the sink position.
SO+CO	$> \frac{2}{3}$ of all G^3 containing a given sector contain it in the source or conduit positions and $> \frac{1}{3}$ of the times in each
SO+SI	$> \frac{2}{3}$ of all G^3 containing a given sector contain it in the source or sink positions and $> \frac{1}{3}$ of the times in each
SI+CO	$> \frac{2}{3}$ of all G^3 containing a given sector contain it in the sink or conduit positions and $> \frac{1}{3}$ of the times in each

Supplementary Information

Sensitivity analysis

We investigated the effects of variating the thresholds used in Methods.

Multiplicative ownership of 0.001: We calculated the sink-OFCs and conduit-OFCs using thresholds for the multiplicative ownership equal to 0.1 and 0.01 (Figure S2). For the threshold of 0.1 two small sink-OFCs (Nauru and Monaco) fell out of this category, and three small sink-OFC were found (Aruba, Guernsey and Saint Kitts and Nevis. Figure S2A). A new small conduit-OFC was also found (Austria. Figure S2B). For the threshold of 0.01 we found the same classification of territories into sink and conduit-OFCs that we found using our original threshold (0.001), which indicates that we achieved convergence (Figure S2C–D). Further lowering the threshold would not provide new benefits and would significantly increase computational time.

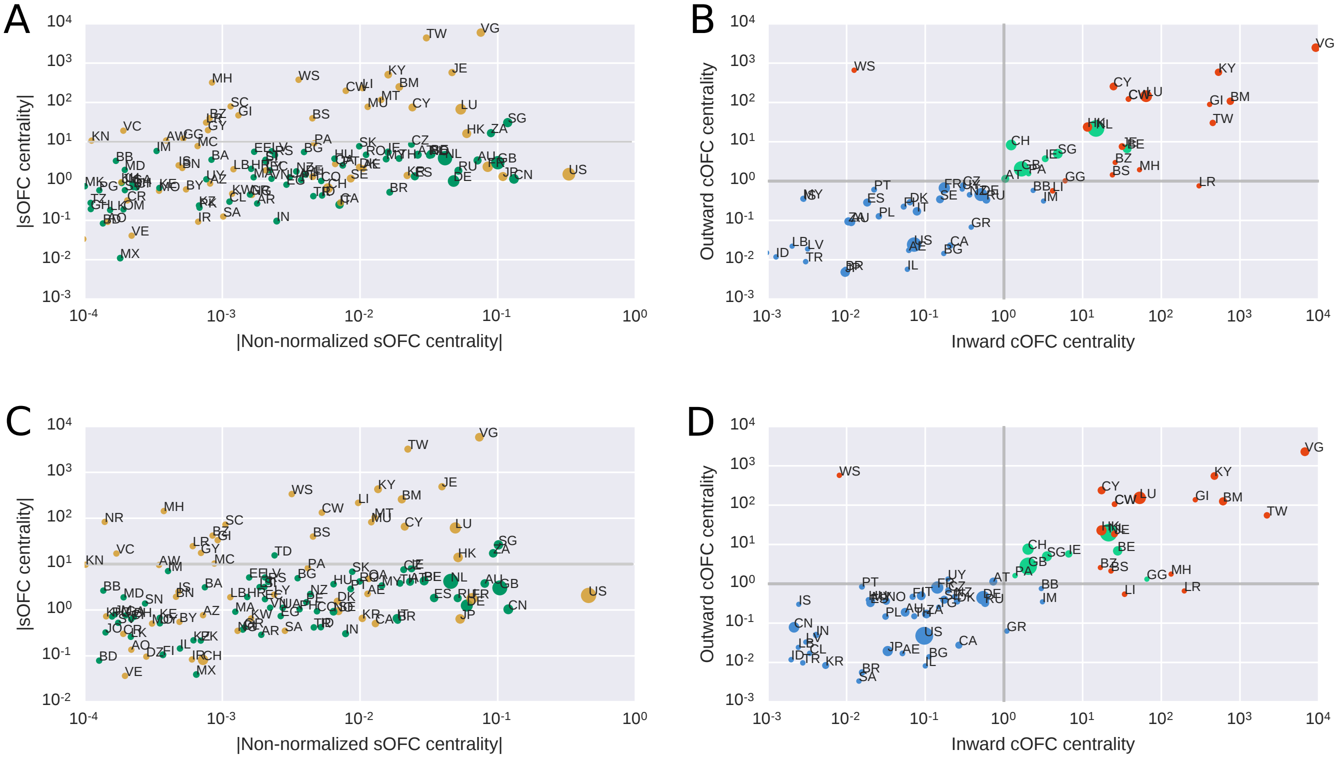

Figure S2. Variation of chain value for different multiplicative ownership thresholds. sink-OFCs (orange) and conduit-OFCs (green) for threshold 0.1 (A–B) and 0.01 (C–D).

$S_c > 10$: We classified countries as sink-OFCs when the value remaining in the country was larger than ten times the GDP of the country ($S_c > 10$). The sink-OFC classification varies with the S_c threshold as reflected in Table S3. The countries identified as conduit-OFCs vary with the S_c threshold as reflected in Table S4. Importantly, the five large conduit-OFCs are found independently of the S_c threshold studied (Table S4). When the S_c threshold is increased to 100, several sink-OFCs (Luxembourg, Cyprus, Hong Kong, Marshall Islands, Gibraltar and Bahamas) become conduit-OFCs (Table S4 and Fig. S3), which indicates a double role of those jurisdictions as sink and conduit-OFCs.

Table S3. sink-OFCs for different thresholds of S_c

Country	$S_c \cdot GDP$	S_c
Virgin Islands, British	$9.4 \cdot 10^{11}$	5235.2
Taiwan, Province of China	$2.3 \cdot 10^{11}$	2277.4
Jersey	$4.6 \cdot 10^{11}$	397.3
Bermuda	$4.1 \cdot 10^{11}$	374.0
Cayman Islands	$1.5 \cdot 10^{11}$	330.7
Samoa	$3.7 \cdot 10^{10}$	276.4
Liechtenstein	$1.4 \cdot 10^{11}$	225.3
Curaçao	$6.5 \cdot 10^{10}$	114.6
Marshall Islands	$3.7 \cdot 10^{9}$	99.6
Malta	$1.7 \cdot 10^{11}$	99.3
Mauritius	$1.6 \cdot 10^{11}$	75.3
Luxembourg	$8.1 \cdot 10^{11}$	71.1
Nauru	$1.6 \cdot 10^{9}$	67.2
Cyprus	$2.8 \cdot 10^{11}$	62.1
Seychelles	$1.2 \cdot 10^{10}$	59.7
Bahamas	$6.5 \cdot 10^{10}$	39.8
Belize	$1.1 \cdot 10^{10}$	37.5
Gibraltar	$1.3 \cdot 10^{10}$	33.8
Anguilla	$9.3 \cdot 10^{8}$	26.8
Liberia	$6.2 \cdot 10^{9}$	17.5
Saint Vincent and the Grenadines	$2.0 \cdot 10^{9}$	14.3
Guyana	$8.1 \cdot 10^{9}$	14.1
Hong Kong	$7.4 \cdot 10^{11}$	14.0
Monaco	$1.3 \cdot 10^{10}$	10.7
Saint Kitts and Nevis	$1.2 \cdot 10^{9}$	8.3
Aruba	$4.0 \cdot 10^{9}$	7.7
Panama	$5.1 \cdot 10^{10}$	7.1
Qatar	$2.3 \cdot 10^{11}$	6.6
Norway	$3.4 \cdot 10^{11}$	3.4
Vanuatu	$5.0 \cdot 10^{8}$	3.2
San Marino	$6.9 \cdot 10^{8}$	3.0
Saint Lucia	$6.7 \cdot 10^{8}$	2.8
United Arab Emirates	$1.8 \cdot 10^{11}$	2.6
Libya	$3.9 \cdot 10^{10}$	2.5
Dominica	$2.3 \cdot 10^{8}$	2.4
United States	$7.2 \cdot 10^{12}$	2.3
Iceland	$6.1 \cdot 10^{9}$	2.3
Brunei Darussalam	$5.6 \cdot 10^{9}$	1.7
Lebanon	$1.3 \cdot 10^{10}$	1.6
Canada	$4.6 \cdot 10^{11}$	1.3
Andorra	$7.7 \cdot 10^{8}$	1.2
France	$5.7 \cdot 10^{11}$	1.1

$C_{c(in/out)} > 1$ We classified countries as conduit-OFCs when the value going through the country into (out) of a sink-OFC was larger than the GDP of the country ($C_{c(in/out)} > 1$). The countries identified as conduit-OFCs are sensitive to changes in the $C_{c(in/out)}$ threshold. For instance moving the threshold from 1 to 0.1 would include a large set of countries into the conduit-OFC category (e.g., France, Germany, Norway, Russia). Moving the threshold from 1 to 10 would make The Netherlands and

Table S4. conduit-OFCs for different thresholds of S_c

Country	Threshold 1	Threshold 10	Threshold 100
Netherlands	conduit	conduit	conduit
Belgium	conduit	conduit	
Switzerland	conduit	conduit	conduit
Guernsey	conduit	conduit	
Singapore	conduit	conduit	conduit
Ireland	conduit	conduit	conduit
United Kingdom	conduit	conduit	conduit
Panama	sink	conduit	
Luxembourg	sink	sink	conduit
Cyprus	sink	sink	conduit
Hong Kong	sink	sink	conduit
Marshall Islands	sink	sink	conduit
Gibraltar	sink	sink	conduit
Bahamas	sink	sink	conduit
Barbados			conduit

Belgium to be the only countries identified as conduit-OFCs (Figure S3C). However, we hypothesized that the set of identified conduit-OFCs constitute a homogeneous cluster. In order to test this, we clustered the territories using the KMeans algorithm from the *sklearn* Python package. We found that all big five conduit-OFCs are always found in the same cluster when we asked the algorithm to find two to six clusters (Figure S4). Moreover, Austria, Panama, Isle de Man, and Barbados are also often in the same cluster than the conduit-OFCs, which is expected since have been considered tax havens. We found that a group of countries composed by The Netherlands, Belgium, Ireland, Singapore, United Kingdom and Switzerland always constitute their own cluster with threshold $C_{c(in/out)} = 1$. This cluster is different from the cluster of sink-OFCs (higher values of C_c) and the cluster(s) of other countries (lower values of C_c). Thus, we found that the division between conduit-OFCs and other countries occur naturally around $C_{c(in/out)} = 1$.

Euroclear and Belgium as a conduit-OFC, Panama and Guernsey

From the set of conduit-OFCs the peripheral position of Belgium stands out. Closer inspection of the underlying data reveals that Belgium derives its conduit-OFC status foremost from the ownership chains $SHELL\ NL \rightarrow Euroclear\ NL \rightarrow Euroclear\ BE \rightarrow Euroclear\ LU$ (Euroclear is a large custodian, which means that in this case there are no data available on the ultimate owners of this stake in Shell). Two other peripheral conduit-OFCs are Panama and Guernsey, since many GCOCs going to sink-OFCs go through the countries in comparison to their GDP. However, both jurisdictions are very small actors.

Comparison of sink-OFC and conduit-OFC centrality with other rankings of offshore financial centers and tax havens

We compared our ranking (based on the value entering the sink) of offshore financial centers to previous rankings and lists of countries (Table S5): (I) Oxfam2016[2], a semi-quantitative assessment of jurisdictions based on the following criteria: “Relatively large role as a corporate tax haven; Corporate Income Tax rate as a proportion of the global average rate; No withholding tax (law, not tax treaties); Aggressive tax planning indicators – score for tax incentives; Lack of Controlled Foreign

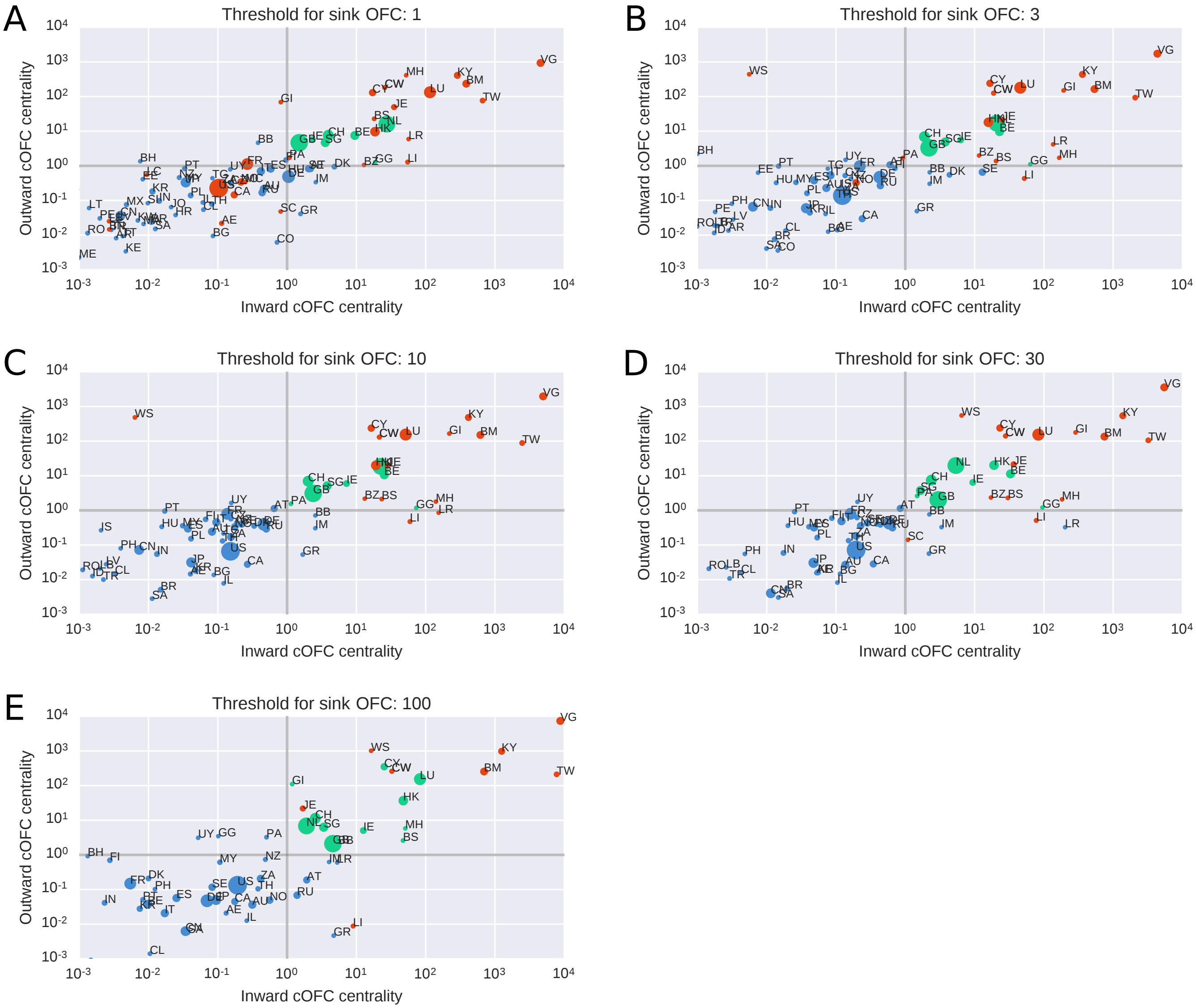

Figure S3. Sink (red) and conduit-OFCs (green) for different threshold of S_c: (A) 1, (B) 3, (C) 10, (D) 30 and (E) 100.

Company rules – CFC; Lack of commitment to international efforts against tax avoidance.” (II) FSI2015[3], a quantitative assessment of jurisdictions based on the secrecy index (a sum of 15 indicators correlated to financial secrecy) and the weight of the jurisdiction in the global trade of financial services. (III) EU2015[4], a simple list released by the European Union. (IV) IMF2000[5], a qualitative assessment based on regulatory framework of the jurisdictions[6]. (V) IMF2008[7], based on the 46 jurisdictions invited to cooperate with the Information Framework[7]. (VI) Fichtner[8], a quantitative approach based on the ratio of the external capital in a jurisdiction with its gross domestic product.

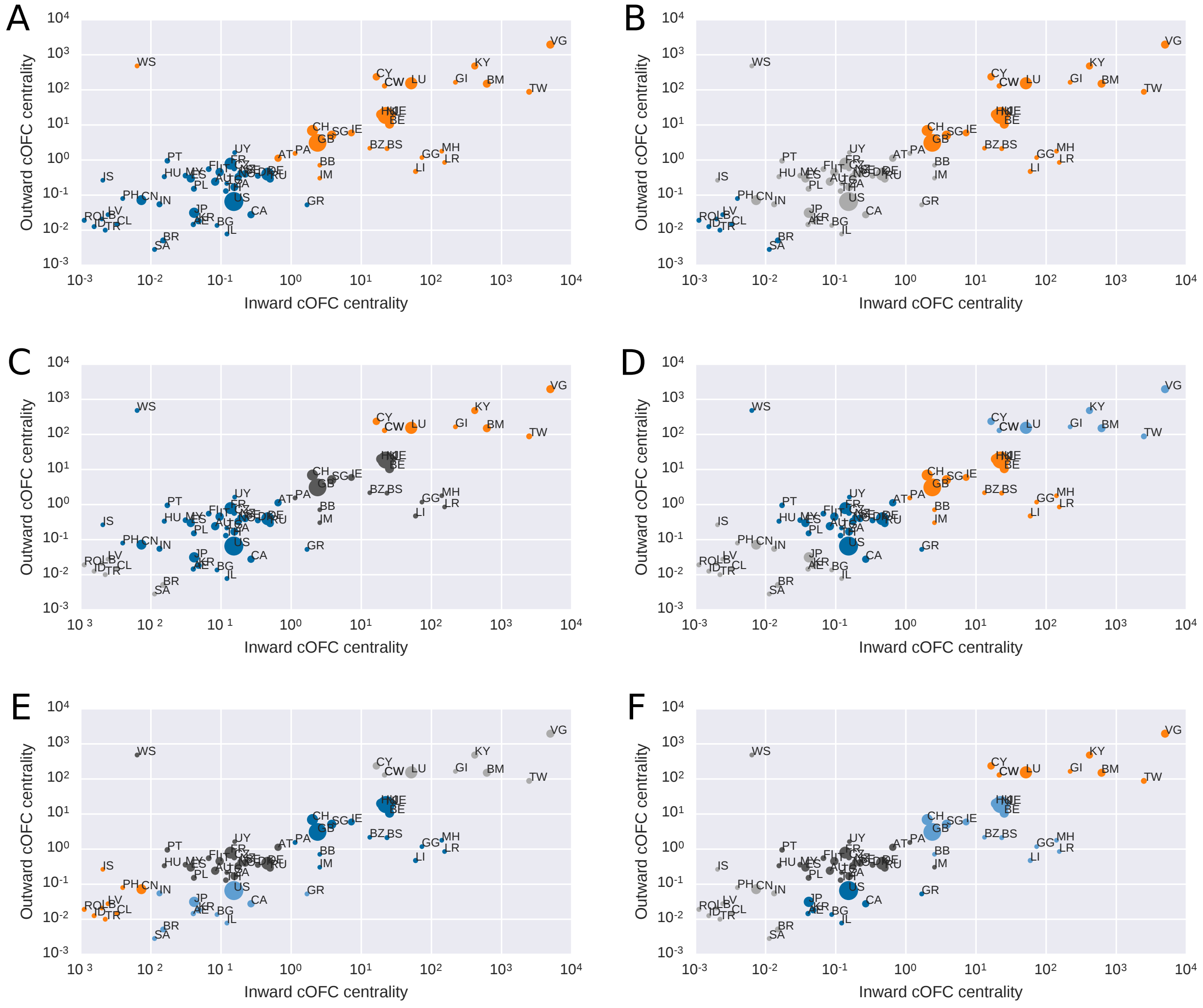

Figure S4. Clusters found using the KMeans algorithm for (A) 2, (B) 3, (C) 4, (D) 5 and (E) 6 clusters. Note that a group of countries formed by among others The Netherlands, Ireland, Singapore, United Kingdom and Switzerland appear always in the same cluster.

Table S5. Comparison of different rankings of countries. 'Dest.' corresponds to the value flowing into the jurisdiction. NN S_c corresponds to the non-normalized sink centrality.

IMF2000 categories; 1: Non-cooperative 2: Below international standards 3: Generally cooperative. *The centrality of Belgium is based on an incorrect classification of one company by the data provider (see Supplementary Information)

	This study	Indicators		$Oxfam_{16}$	FSI_{15}	EU_{15}	IMF_{00}	IMF_{08}	Fichtner
	sink-OFC	Dest.	NN S_c						
Luxembourg	1	$3.2\cdot10^{12}$	$8.1\cdot10^{11}$	7	6		3	x	5
Hong Kong	2	$1.9\cdot10^{12}$	$7.4\cdot10^{11}$	9	2		3	x	14
British Virgin Isl.	3	$1.3\cdot10^{12}$	$9.4\cdot10^{11}$	15	21	x	1	x	1
Bermuda	4	$1.1\cdot10^{12}$	$4.1\cdot10^{11}$	1	34	x	2	x	3
Cyprus	5	$8.9\cdot10^{11}$	$2.8\cdot10^{11}$	10	35		1	x	7
Cayman Islands	6	$7.3\cdot10^{11}$	$1.5\cdot10^{11}$	2	5	x	1	x	2
Jersey	7	$5.5\cdot10^{11}$	$4.6\cdot10^{11}$	12	16		3	x	11
Taiwan	8	$3.8\cdot10^{11}$	$2.3\cdot10^{11}$						
Malta	9	$1.7\cdot10^{11}$	$1.7\cdot10^{11}$		27		2	x	
Mauritius	10	$1.6\cdot10^{11}$	$1.6\cdot10^{11}$	14	23		1		8
Liechtenstein	11	$1.6\cdot10^{11}$	$1.4\cdot10^{11}$		36		1	x	
Curaçao	12	$1.5\cdot10^{11}$	$6.5\cdot10^{10}$	8	70		1	x	6
Bahamas	13	$9.2\cdot10^{10}$	$6.5\cdot10^{10}$	11	25	x	1	x	9
Samoa	14	$5.7\cdot10^{10}$	$3.7\cdot10^{10}$		51		1	x	4
Gibraltar	15	$4.9\cdot10^{10}$	$1.3\cdot10^{10}$		55		2	x	12
Marshall Islands	16	$2.3\cdot10^{10}$	$3.7\cdot10^{9}$		14		1	x	
Monaco	17	$1.5\cdot10^{10}$	$1.3\cdot10^{10}$		76	x	2	x	
Liberia	18	$1.4\cdot10^{10}$	$6.2\cdot10^{9}$		33		x		
Seychelles	19	$1.2\cdot10^{10}$	$1.2\cdot10^{10}$		72		1	x	
Belize	20	$1.2\cdot10^{10}$	$1.1\cdot10^{10}$		60	x	1	x	
Guyana	21	$8.1\cdot10^{9}$	$8.1\cdot10^{9}$						
St Vincent & Gren.	22	$2.2\cdot10^{9}$	$2.0\cdot10^{9}$		64	x	1	x	
Nauru	23	$1.6\cdot10^{9}$	$1.6\cdot10^{9}$				1	x	
Anguilla	24	$1.0\cdot10^{9}$	$9.3\cdot10^{8}$		63	x	1	x	
	conduit-OFC	Non normalized $\overline{C_c}$							
Netherlands	1	$5.3\cdot10^{11}$		3	41				15
United Kingdom	2	$2.2\cdot10^{11}$			15		x		21
Switzerland	3	$7.9\cdot10^{10}$		4	1		3	x	17
Ireland	4	$4.6\cdot10^{10}$		6	37		3	x	16
Singapore	5	$4.0\cdot10^{10}$		5	4		3	x	20
Belgium*	Small	$2.6\cdot10^{11}$			38				19
Panama	Small	$1.6\cdot10^{9}$			13	x	1	x	
Guernsey	Small	$9.6\cdot10^{8}$			17	x	3	x	10
	non-OFCs								
Barbados				13	22	x	2	x	13
Antigua & Barbuda					65	x	1	x	
Grenada					82	x	x	x	
Montserrat					92	x	x	x	
St. Kitts and Nevis					69	x	1	x	
Turks & Caicos Isl.					68	x	1	x	
US Virgin Islands					50	x			

References

1. Garcia-Bernardo, J. & Takes, F. W. The effects of data quality on the analysis of corporate board interlock networks. *arXiv preprint arXiv:1612.01510* (2016).
2. Berkhout, E. Tax battles: The dangerous global race to the bottom on corporate tax. Tech. Rep., Oxfam policy paper (2016).
3. Cobham, A., Jansky, P. & Meinzer, M. The financial secrecy index: Shedding new light on the geography of secrecy. *Economic geography* **91**, 281–303 (2015).
4. EUBusiness. *EU releases world tax havens blacklist* (2015).
5. International Monetary Fund. *Offshore Financial Centers: IMF Background Paper* (IMF, 2000).
6. Errico, M. L. & Borrero, M. A. M. *Offshore banking: an analysis of micro-and macro-prudential issues* (IMF, 1999).
7. International Monetary Fund. *Offshore Financial Centers: A Report on the Assessment Program and Proposal for Integration with the Financial Sector Assessment Program* (2008).
8. Fichtner, J. The offshore-intensity ratio. identifying the strongest magnets for foreign capital. *CITYPERC Working Paper Series 2015/02, City University London. SSRN ID 2928027* (2015).
